# Factors affecting asthma control in pregnancy: A cross-sectional study

**DOI:** 10.12669/pjms.40.6.8659

**Published:** 2024-07

**Authors:** Kaleem Ullah Toori, Muhammad Arsalan Qureshi

**Affiliations:** 1 Kaleem Ullah Toori, FRCP, Department of Medicine, KRL Hospital, Islamabad, Pakistan; 2 Muhammad Arsalan Qureshi, MBBS, Department of Medicine, KRL Hospital, Islamabad, Pakistan

**Keywords:** Asthma, Pregnant women, Parity, BMI, Inhaler technique, Treatment compliance

## Abstract

**Background and Objective:**

Asthma control in pregnant women remains of utmost importance; suboptimal control can have adverse repercussions on both fetal and maternal health. The objective was to identify the factors that affect asthma control in pregnant Pakistani women presenting to a tertiary care hospital.

**Methods:**

This descriptive, cross-sectional research was conducted at KRL General Hospital between 1^st^ November 2022 to 30^th^ April 2023. Non-probability technique was used to sample one hundred and forty-five pregnant women with confirmed bronchial asthma irrespective of their trimester presented. Data regarding demographics and factors affecting asthma control was collected.

**Results:**

The mean age was 30.39 ± 4.33 years, with two-thirds (65%) being multiparous. Approximately 48% of participants were non-compliant with treatment, and less than 40% achieved adequate asthma control. A chi-squared test applied showed that multiparity (p = 0.003), treatment compliance (p < 0.001), BMI (p < 0.001), and proper inhaler technique (p < 0.001) were statistically significant factors affecting asthma control in pregnant women while, the level of education and household income did not exhibit a significant association. Multiple regression analysis qualified higher BMI, multiparity, treatment compliance, and inhaler technique as significant predictors of asthma control amongst pregnant women.

**Conclusion:**

Ensuring asthma control during pregnancy is important. This study identified BMI, multiparity, inhaler technique, and treatment compliance as factors that affect asthma control in pregnant women. Addressing these factors through regular antenatal check-ups can significantly mitigate risks and promote the optimal health of both maternal and fetal lives.

## INTRODUCTION

Asthma is characterized by chronic airway inflammation with respiratory symptoms, including chest tightness and wheezing with variable airflow limitation.^1^ It is a major healthcare problem that continues to affect millions of people worldwide including all age groups. In 2019 over 250 million people were affected with over 450,000 deaths.^2^

A 15-years-old cohort study of women in their productive age from the United States reported asthma prevalence of 10.9% in pregnant women.^3^ A regional Study conducted in Karachi, Pakistan, found the prevalence to be between 1.8% to 11%,^4^ which included both sexes. A study done by Fazel et al found well-controlled asthma and favorable quality of life to be 38% and 5.8% respectively.^5^

The standard care for Asthma involves inhaled beta-agonists and corticosteroids, leukotriene antagonists, and biologic agents in difficult-to-treat cases.^1^ The course of asthma during pregnancy is acknowledged to demonstrate diversity throughout the various stages of pregnancy.^6^ Insufficient control of asthma can significantly impact patients’ quality of life, imposing limitations on their daily activities.^5^ Extensive research has consistently highlighted the heightened vulnerability of pregnant women with asthma to develop hypertension in pregnancy^7^, preeclampsia^7^, gestational diabetes^8^ and an increased risk of contracting a urinary tract infection.^9^ Fetal complications include post-birth Neonatal ICU admission, small for gestational age, low birth weight^10^, prematurity birth asphyxia^11^, and an increased risk of having congenital malformations.^7^ Maternal Asthma leads to an increased risk of childhood Asthma and Pneumonia.^7^

Literature search has identified covariates that can adversely affect asthma control, which include age, parity, obesity, socioeconomic status, education, and smoking.^12^ Also, asthma exacerbation during pregnancy can adversely affect the perinatal and infant health outcomes.^13^ By monitoring pregnant women with asthma, healthcare providers can promptly identify and take proactive measures to mitigate the impact of asthma exacerbations and reduce maternal and fetal complications.^7^

This study aims to identify the factors influencing asthma control in pregnant women within the Pakistani population. To the best of our knowledge, this is the first local study of its kind that addresses this clinical dilemma, providing valuable guidance at a local level on effectively managing the burden of maternal asthma.

## METHODS

The research was conducted at KRL General Hospital, which is a 350 bedded teaching hospital with dedicated Pulmonology and OB/GYN clinical units. The duration of the study was six months from 1^st^ November 2022 to 30^th^ April 2023

### Ethical Approval

Before collecting data, permission was taken from the local ethics committee with the following reference number ‘KRL-HI-PUB-ERC/29 dated 15-12-202’. Informed consent was obtained from all participants.

The study design was a descriptive cross-sectional. The sampling technique was nonprobability convenience sampling. The sample size was calculated using the WHO samples size calculator. The sample size of the target population was 145 with a confidence interval of 95% and absolute precision of 8% with a prevalence of well-controlled asthma of 38%.^5^

### Inclusion & Exclusion Criteria

All pregnant women with asthma, regardless of the trimester of pregnancy, were eligible to participate. It is essential that the asthma diagnosis be confirmed by a pulmonologist and supported by spirometry with reversibility testing (an increase in FEV1 of ≥ 12% or 200mls after bronchodilator challenge) as per Global Initiative for Asthma (GINA) guidelines.^1^ On the other hand, pregnant ladies with self-reported asthma without physician confirmation and with other obstructive lung diseases like COPD and Bronchiectasis were excluded.

Data was collected from patients presenting to the Department of OB/GYN and Pulmonology with a history of asthma who were expected to deliver at KRL hospital. To assess asthma control, an Asthma symptom control questionnaire outlined by GINA^1^ was used and frequency of daytime symptoms, night-time awakening, short-acting beta-agonist use, and exercise limitation, to assess the patient’s asthma control were recorded.

### Statistical Analysis

Data was collected and entered in SPSS version 25. Quantitative variables like age, GINA assessment question score, and duration of asthma were recorded as mean and SD values. Mean ± SD were reported for the normally distributed (Shapiro Wilk test) while the median (IQR) was reported for the non-normality distributed quantitative variables. Frequencies and percentages were calculated for categorical variables like residence status, parity, gravida, family monthly income, occupational status, educational status.

Effect modifiers were controlled through stratification of age, residence status, parity, gravida, family monthly income, occupational status, educational status, and duration of asthma to see the effect of these on the outcome variable. Post-stratification Chi-square test was applied and a p-value of ≤ 0.05 was considered significant. To assess the potential relationship between Asthma control and the factors influencing it, a Spearman correlation analysis was employed. This analysis aimed to determine whether a significant association existed between Asthma control and the aforementioned factors. The multivariate regression model was to used examine the impact of variables on asthma, considering their combined effects.

## RESULTS

A total of 145 pregnant women were included in this study irrespective of the trimester who were booked at KRL Hospital. The participants mean age was 30.39 years (SD = 4.33), ranging from 18 to 45 years. The average duration of Asthma was 5.39 years with an interquartile range (IQR) of 4.00 to 7.00. The mean GINA assessment score was 1.64 with an IQR of 0.00 to 3.00.

Majority of the patients, 65% (n=95), were multiparous, 54.5% of the participants (n=79) lived in rural areas and 67.65% (n=98) were unemployed. Regarding education level, approximately 43% completed a bachelor’s degree, while the remaining were educated up to grade 12 or less (Table-I).

**Table-I:** Baseline characteristics of study population.

Factors	Frequency	Percentage
Duration of Asthma	5.39 ± 2.27*	
Age	30.39 ± 4.33*	
***Residence***		
Rural	79	54.5
Urban	66	45.5
***Parity***		
Primiparous	50	34.5
Multiparous	95	65.5
***Employment Status***		
Employed	47	32.4
Unemployed	98	67.6
***Compliance***		
Compliant	76	52.4
Non-Complaint	69	47.6
***GERD***		
Yes	69	47.6
No	76	52.4
***BMI***		
<18.5	13	9.0
18.6-24.9	88	60.7
25.0-29.9	36	24.8
>30.0	8	5.5
***Income***		
<50,000	59	40.7
>50,000	86	59.3
***Education***		
Illiterate	24	16.6
Primary	20	13.8
Secondary	38	26.2
Graduate	63	43.4
***Inhaler Technique***		
Inadequate	88	60.7
Adequate	57	39.3
***Gina Control***		
Uncontrolled	42	29.0
Partly Controlled	50	34.5
Controlled	53	36.6
***Smoker***		
Yes	0	0
No	145	100

* Mean with Standard Deviation.

A chi-squared test applied showed that multiparity (p = 0.003), treatment compliance (p < 0.001), BMI (p < 0.001), and proper inhaler technique (p < 0.001) were statistically significant factors affecting asthma control in pregnant women. However, the level of education and household income did not exhibit a significant association with asthma control in our study population.

Spearman correlation was utilized to examine the relationship between asthma control and other variables. Poor compliance (Spearman’s r = - 0.422, p<0.001), poor inhaler technique (Spearman’s r = -0.71, p <0.001) and higher BMI (Spearman’s r = -0.459, p <0.001) were associated with poor asthma control while multiparity was associated with better control (Spearman’s r = 0.29, P=0. 001) in pregnant women.

Multiple regression analysis qualified higher BMI, multiparity, treatment compliance, and inhaler technique as significant predictors of asthma control amongst pregnant women [F (9.135) =18.924 p < 0.001 R^2^= 0.558]. The standardized Beta Coefficient is also reported for each variable in the regression model in Table-IV.

**Table-II:** Association of individual factors with GINA Score.

Factors	Value	DOF	p value
Duration of Asthma	18.57	16	0.291
Age	28.14	24	0.254
Residence	0.106	2	0.948
Parity	11.64	2	0.003
Employment Status	1.08	2	0.581
Compliance	31.43	2	<0.001
GERD	2.27	2	0.321
BMI	36.02	6	<0.001
Income	1.87	2	0.392
Education	7.32	6	0.292
Inhaler Technique	99.21	2	<0.001

DOF: Degree of Freedom, GINA: Global Initiative for Asthma.

**Table-III:** Overall result of multivariate linear regression analysis between GINA Control and Factors.

ModelR	R Square	Adjusted R Square	Std. Error of the Estimate
0.747	0.558	0.528	0.555

GINA: Global Initiative for Asthma.

**Table-IV:** Multivariate Analysis for Factors Associated with Asthma Control.

Factors	Lower Bound	Upper Bound	β Coefficient	t	p Value
Parity	-0.010	0.418	0.126	2.079	0.04
Employment Status	-0.263	0.131	-0.038	-0.662	0.509
Compliance	-0.447	-0.033	-0.148	-2.289	0.024
GERD	-0.119	0.255	0.042	0.723	0.471
BMI	-0.351	-0.057	-0.176	-2.740	0.007
Income	-0.249	0.147	-0.031	-0.508	0.612
Education	-0.070	0.101	0.021	0.358	0.721
Technique	-1.043	-0.560	-0.486	-6.557	0.000

## DISCUSSION

Bronchial asthma is a common condition and uncontrolled asthma can have deleterious effects on maternal and fetal health during pregnancy.^3^,^7^-^10^ This study has revealed that asthma control was influenced by BMI, multiparity, treatment compliance, and proper inhaler technique. Overall, this points toward the factors that need to be addressed in pregnant women with asthma to prevent poor control.

Interestingly, we found that as the parity increased, our target population showed better asthma control, contrary to the results in studies conducted elsewhere.^14^,^15^ We can attribute this finding to relatively small sample size of our study population and the study design itself; which only measured control at one point in time. Further studies with a higher sample size and ideally a prospective cohort study design may provide more reliable evidence.

Keeping in line with Murphy et al^16^; we also observed association between higher body mass index and inadequate asthma control. It is hereby suggested that women of childbearing age be encouraged to lose weight before planning to conceive. It has been observed that obesity leads to the development of reduced lung volumes, bronchial hyperresponsiveness, and inability to initiate deep breathing movements. It is further complicated by impaired immune function which may contribute to the worsening of asthma in obese patients.^17^

Healthcare professionals managing women with asthma who are pregnant or are planning to conceive should identify people who are at high risk; i.e., smokers, obese, or those who have history of poor treatment adherence.^12^ All these modifiable risk factors should be addressed in order to mitigate the adverse outcome associated with maternal asthma.^13^ In our study, slightly over 50% of our study participants were compliant with their treatment, indicating significant room for improvement. Robijn et al reported 38% of pregnant women being non-compliant to asthma treatment specifically the use of inhaled corticosteroids.^18^ Williams et al concluded that individuals maintaining a compliance rate of 75% would experience notable prevention of severe asthma exacerbations.^19^

Over 60% of our study population had an improper inhaler technique. The majority of individuals are unaware that possessing a suboptimal inhaler technique constitutes a problem. An Italian study^20^ reported up to 44% and an Indian study^21^ reported 37% of the patients having poor inhaler technique. An improper inhaler technique can lead to poor disease control.^20^ Our Study reports a relatively higher proportion with inadequate inhaler technique. We may attribute this to a small sample size in comparison to both these studies. Realistically, a lack of knowledge of correct inhaler technique reflects poor control and it is vital to identify this as simple education about correct technique can lead to improved control and reduced morbidity.

The utilization of inhalation devices allows for the delivery of medication directly to the airways, resulting in a concentrated dose and quicker therapeutic effects, while minimizing systemic side effects.^1^ However, acquiring the appropriate skills for proficient inhaler technique is essential to achieve optimal drug delivery. According to GINA^1^ almost 70 to 80% of patients with asthma have a suboptimal inhaler technique. GINA has recommended the use of placebo inhalers and spacing devices to train patients by demonstration. Additionally, it is advised to periodically reevaluate and retrain patients as necessary.

### Limitations

This study is subject to certain limitations, including its single-center design and relatively small sample size. To gain a more comprehensive understanding of how maternal asthma impacts perinatal and postnatal outcomes, future research should consider conducting similar studies involving multiple centers and employing a prospective cohort study design. Furthermore, following patients throughout the postnatal period would provide valuable insights, enabling a more holistic approach to addressing this issue.

## CONCLUSION

Our study has demonstrated BMI, multiparity, inhaler technique, and treatment compliance as factors that affect asthma control in pregnant women. It is therefore suggested that the modifiable risk factors amongst these may be addressed at every follow-up visit to ensure a better outcome for both the mother and the fetus. It’s essential to highlight that correct inhaler technique and advise for treatment compliance be given and documented with each antenatal visit.
